# The efficiency of the public dental services (PDS) in Cyprus and selected determinants

**DOI:** 10.1186/1472-6963-13-420

**Published:** 2013-10-18

**Authors:** Chrystalla Charalambous, Nikolaos Maniadakis, Nikolaos Polyzos, Vassilis Fragoulakis, Mamas Theodorou

**Affiliations:** 1 Open University of Cyprus, Lemesou Avenoue 2, Aluminium Tower, 2003, Strovolos, Lefkosia, Cyprus; 2 Health Services Organisation & Management, National School of Public Health, 196 Alexandras Avenue, 115 21, Athens, Greece

**Keywords:** Efficiency measurement, Data envelopment analysis, Public dental services, Cyprus

## Abstract

**Background:**

Currently there is a dual system of oral healthcare delivery in Cyprus: the public dental system (PDS) run by the Government and the private system provided by private dental practitioners. Although 83% of the population is entitled to free treatment by the PDS only 10% of the population make use of them. As Cyprus faces now the challenges of the introduction of a new health care system and rising healthcare costs in general, surveys that examine, among other things, the efficiency of the PDS become very important as tools to make important cost savings. The aims of this study are to assess trends regarding the number of visits and the age distribution of patients using PDS from 2004 to 2007, to measure the technical efficiency of the PDS and to investigate various factors that may affect it.

**Methods:**

Non-parametric Data Envelopment Analysis (DEA) was employed to assess technical efficiency. Two separate cases were examined. Efficiency was calculated, firstly using as inputs the wages and the working hours of the personnel, and secondly the working hours of the personnel and the cost of the materials. As outputs, in both cases, the treatment offered (divided into primary, secondary and tertiary care) and the numbers of visits were used. In the second stage Tobit analysis was used to explore various predictors of efficiency (time per patient, location, age of dentists, age of patients and age of assistants).

**Results:**

The study showed that whilst there was an increase in the number of patients using the PDS from 2004 to 2007, only a small proportion of the population (10%) make use of them. Women, middle and older aged patients, make more use of the PDS. Regarding efficiency, there were large differences between the units. The average Technical Efficiency score was 68% in the first model and 81% in the second. Urban areas and low time per patient are predictors of increased efficiency.

**Conclusion:**

The results suggest that many of the rural PDS are underperforming. Given that the option of shutting them down is undesirable, measures should be taken to reduce inputs (e.g. by reducing the personnel’s working hours) and to increase outputs (remove barriers, make PDS more accessible and increase the number of patients).

## Background

Cyprus is the third largest island in the Mediterranean. Currently there is a dual system of oral healthcare delivery in Cyprus: the public dental system (PDS) run by the Government and the private system provided by private dental practitioners. In addition, some dentists have contracts with workers’ unions or other semi-governmental organizations, as well as insurance companies. Payment is usually charged per item of service provided [1,2]. The costs of the PDS are met by the Government. The percentage of the budget given to the PDS is about 1.3% of the entire budget for the Ministry of Health. Of note is the fact that that the proportion of gross domestic product (GDP) spent on healthcare (including dentistry) is only 5.6% [3]. There are 732 dentists in Cyprus, only 40 of them (6%) work in the public sector, and the dentist-to-population ratio is 1 per 1000 [4]. Public Health Service dentists are all salaried and are not permitted to undertake private practice [3]. Public Oral Healthcare is provided at outpatient departments of hospitals, in urban centers and in smaller health centers in rural areas. There are 34 health centers spread all over the country staffed with a dentist and a dental assistant (however in five rural health centers with limited number of patients the dentists work without an assistant). All of them provide services from 7:30 to 14:30. Whilst outpatient departments of hospitals operate on a daily basis, some urban and rural health centers operate three days per week and some operate on a weekly basis. In rural health centers dentists work fewer hours because of reduce demand for dental treatment. The reduced demand is correlated with the number of inhabitants which is gradually decreasing (according to data from the Statistical Service (5) 70% of the population lives in urban areas), the age and the socioeconomic profile of the inhabitants in rural areas as well as to the fact that the younger people in rural areas, chose to visit urban health centers or hospitals which are closer to their work-places. Of note that there are no geographical or any other kind of restrictions regarding visiting a health centre. Therefore, in case of emergency someone may visit the nearest health centre (since Cyprus is a small island the distance between the health centers is no more than 30 minutes) or the district hospitals which have emergency departments staffed with dentists 24 hours a day, 365 days per year.

83% of the population is entitled to free treatment by the Public Dental Services and in that percentage are included: all low income earners (annual income less than €16,000), all government employees (regardless of annual income), schoolchildren and all persons registered as disabled. Everybody else who wishes to be examined and treated by the Public Dental Services has to pay according to fee schedules set by the Ministry of Health. Around 10% of the population accesses dental care through the PDS [5]. A comprehensive spectrum of dental services, including primary, secondary care and tertiary care, is offered by the Public Dental Services in hospitals. In urban and rural health centres only primary and secondary care are offered. Primary care includes all the preventing actions such as examination, fissure sealants and topical application of fluoride, Secondary care represents the main work undertaken in a dental clinic such as fillings, extractions, root canal treatment, while tertiary care is taking place only at hospitals and includes surgical operations under local anaesthesia by a specialist maxillofacial surgeon as well as the construction of removable dentures, a procedure that requires the presence of a dental laboratory. An orthodontics service, however, is not currently provided by the PDS [3].

Until now, the only measurement of the performance of PDS was the number of people attending the service. However, as Cyprus faces the challenges of the introduction of a new health care system and rising healthcare costs in general, surveys that examine, among other things, the efficiency of the PDS become very important as tools to improve the quality of care, to meet the increasingly demanding needs and expectations of the public in Cyprus and to make important cost savings in doing so. An understanding of economic evaluation and efficiency is important for the purchasers, as well as for the providers and financers.

This is the first study that has been conducted, not only by the Public Dental Services, but generally by the Medical Services in Cyprus to examine technical efficiency. The results will form a baseline for future studies of on the PDS and health services assessment in Cyprus. Specifically, the aim of this study was to examine: i) changes regarding the number of visits and the offered treatment by the PDS in Cyprus from 2004 to 2007; ii) the efficiency of the PDS during 2006; and iii) predictors of efficiency.

## Methods

**Figure 1:**
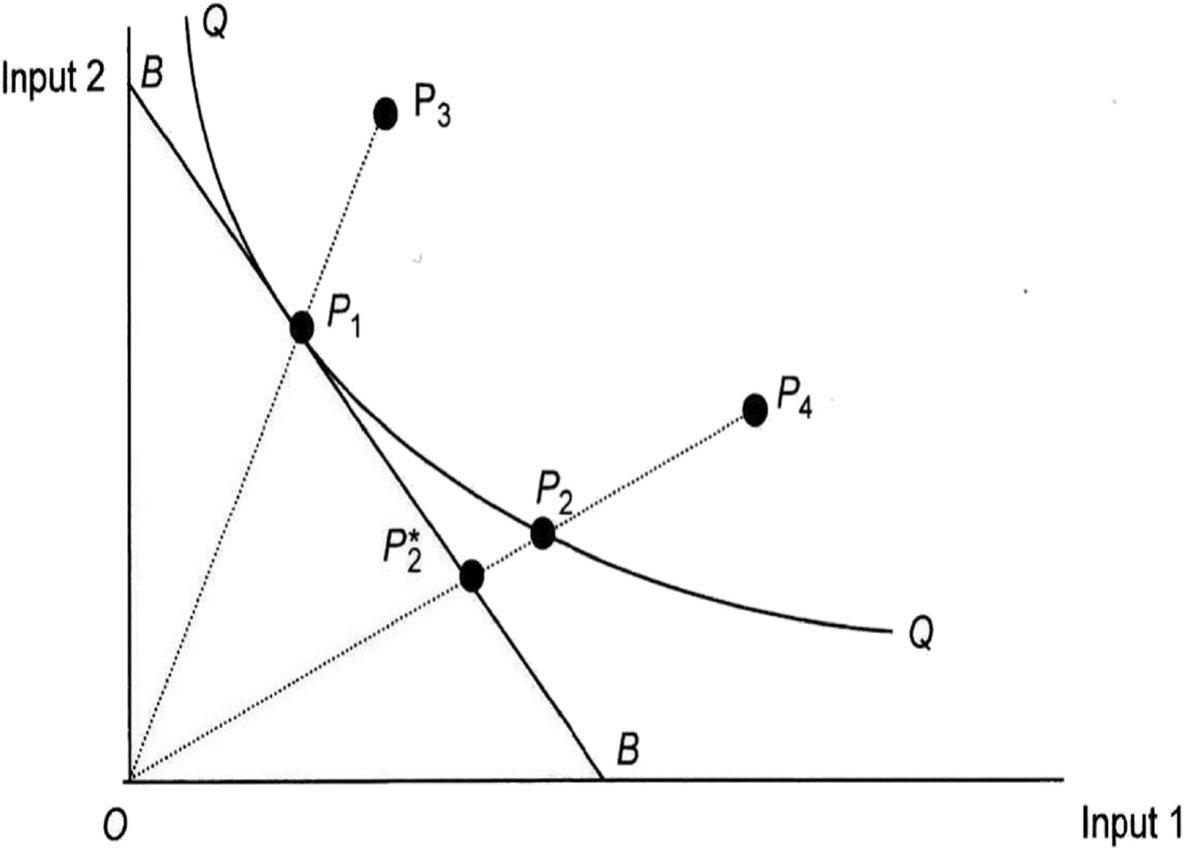
Input oriented efficiency measurement.

Efficiency measurement deals with ways to measure and analyse the productive performance of production units. Following the seminal work of Farrell in 1953 [6], from an input perspective, an organization is technically efficient if output is produced with the minimum possible amount of input, whilst it is allocatively efficient if inputs are also employed in the correct proportion in terms of their prices to minimize production cost for a given output produced and these two concepts define overall efficiency [7]. To illustrate these concepts graphically, consider the simple case of a single output (y) being produced from two inputs (x1) and (x2) in Figure 1. All units on the efficiency frontier QQ operate efficiently by producing the same output with different combinations but always minimal amounts from the two inputs. The line that goes through BB is the minimum isocost line. Unit P_1_ is both technically and allocatively efficient since it uses both inputs optimally given their relative prices. Unit P_2_, although technically efficient as it lies on the frontier, is allocativelly inefficient since it fails to minimize cost in the production of output. Unit P_4_ is both technically inefficient (the technical efficiency score is captured by the ratio OP_2_/OP_4_) and alloctaively inefficient (allocative efficient is defined by the ratio OP^*^_2_/OP_2_). The overall efficiency for unit P_4_ can be found by the multiple of technical and allocative efficiency. Finally unit P_3_ is allocatively efficient but technically inefficient, the later captured by the ratio OP_1_/OP_3_.

A commonly used methodology employed in efficiency measurement is that of Data Envelopment Analysis (DEA) [7]. DEA has several strengths. As a non parametric method it does not require knowledge of the underlying production function, it can easily handle multiple inputs and outputs and has the ability to identify easily actual good practice and performance targets. For these reasons it is the most frequently employed method for measuring the productive performance of health care services. Hence, in the present study, a non-parametric DEA approach was firstly employed to assess technical efficiency.

In DEA there is an issue regarding which inputs and outputs to employ; there are no tests that can aid this process, as there are in the context of the econometric approach. For this reason two separate input–output sets were analysed. As indicated in Table 1, in the first case efficiency was calculated using as inputs the working hours of the personnel and their wages (the wages of the personnel represent 80% of the total costs of the budget of the PDS and are mostly related with the years of experience) and in the second case the working hours of the personnel and the cost of the materials. A lot of variables couldn’t be used at the same time as the number of the examined health centres was small (only 34). As a rule of thumb the examined units should be at least three times more than the examined variables, otherwise there is the risk of fictitious increase of the efficiency [8]. Other variables such as the extent of the hospital facilities and the number of hospital beds weren’t used as the specific range of the offering services by the PDS doesn’t need in patient care and also because regardless of the extension of the facilities, each health centre has only one dental unit.

**Table 1:** Input and output variables

	***Input***	***Output***
Model 1	Working hours of dentists	Primary dental care
	Working hours of assistants	Secondary dental care
	Working hours of technicians	Tertiary dental care
	*Wages of dentists*	Number of visits
	*Wages of assistants*	
	*Wages of technicians*	
Model 2	Working hours of dentists	Primary dental care
	Working hours of assistants	Secondary dental care
	Working hours of technicians	Tertiary dental care
	*Costs of materials*	Number of visits

The outputs considered in both cases include the treatment offered (disaggregated as primary, secondary and tertiary care) and the number of visits. The allocation of treatment to primary, secondary and tertiary was necessary in order to take into consideration the different complexity of the treatment offered. This is a sort of case-mix adjustment. It can be argued that the lack of a more sophisticated case-mix measure for dental care is not that problematic, compared for instance with specialized hospital care, as the variation of resource use and the complexity of care across patient cases is not significant.

In the second part of the study, the estimated efficiency scores were regressed against a set of observed variables which could potentially influence performance. These include the location (urban versus rural to account for accessibility and population demographics^a^), time per patient (to account for quality of care), age of dentist and age of assistants (to account for experience of the team) and age of patients (to account for the nature of the case). In the PDS there are five health centres where the dentists work without any assistant. The relation of the exogenous variables with technical efficiency was examined using a Tobit model. This approach is preferred to the linear regression approach because the dependent variables are censored, and because DEA efficiency scores are continuous on the 0 to 1 interval and take the value 1 with positive probability, while the probability of obtaining the limiting value 0 is zero. All the necessary data regarding working hours of the personnel, wages, cost of the materials, offered treatment (disaggregated as primary, secondary and tertiary), number of visits and also about time per patient and age of dentists, assistants and patients were collected from the annual internal reports of the Public Dental Services (PDS) and were used for the development of this study with the written permission of the Director of the PDS. (Table 2 presents the working hours of the personnel as well as their wages/salaries divided by the type of the health centre while Table 3 presents the offering care divided again by the type of the health centre. The FRONTIER software was used to compute efficiency scores and to identify best practice units and the software STATA ver 8.0 was used for the Tobit model.

**Table 2:** The working hours and the wages of the personnel working at the PDS

**Type of health centre**	**Working hours (hours)**			**Wages (€)**		
	**Dentists**	**Dental assistants**	**Dental technicians**	**Dentists**	**Dental assistants**	**Dental technicians**
Hospitals	41111,00	46692,00	15592,00	551722,03	346385,86	125769,01
Urban	5588,00	5588,00	-	80704,77	42520,96	-
Rural	6033,44	6033,44	-	81409,85	33959,96	-

**Table 3:** The number of care and the offering care by the PDS divided by type of care and type of health centre

**Type of health centre**	**Type of Care (No of actions)**			**No of visits**
	**Primary dental care**	**Secondary dental care**	**Tertiary dental care**	
Hospitals	7554	44991	4884	62310
Urban	2133	17075	0	14516
Rural	1788	8657	0	8963

Ethical approval for the conduction of this study was given by the Public Dental Services of the Ministry of Health in Cyprus.

## Results

**Figure 2:**
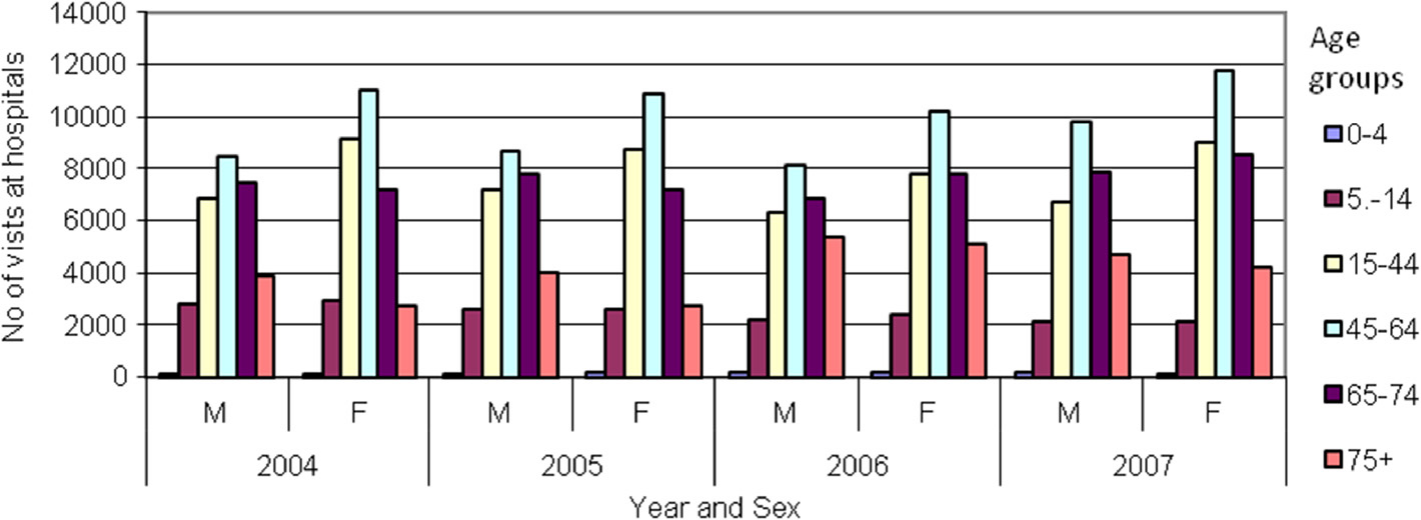
Visits in hospital dental departments by age, sex and year.

**Figure 3:**
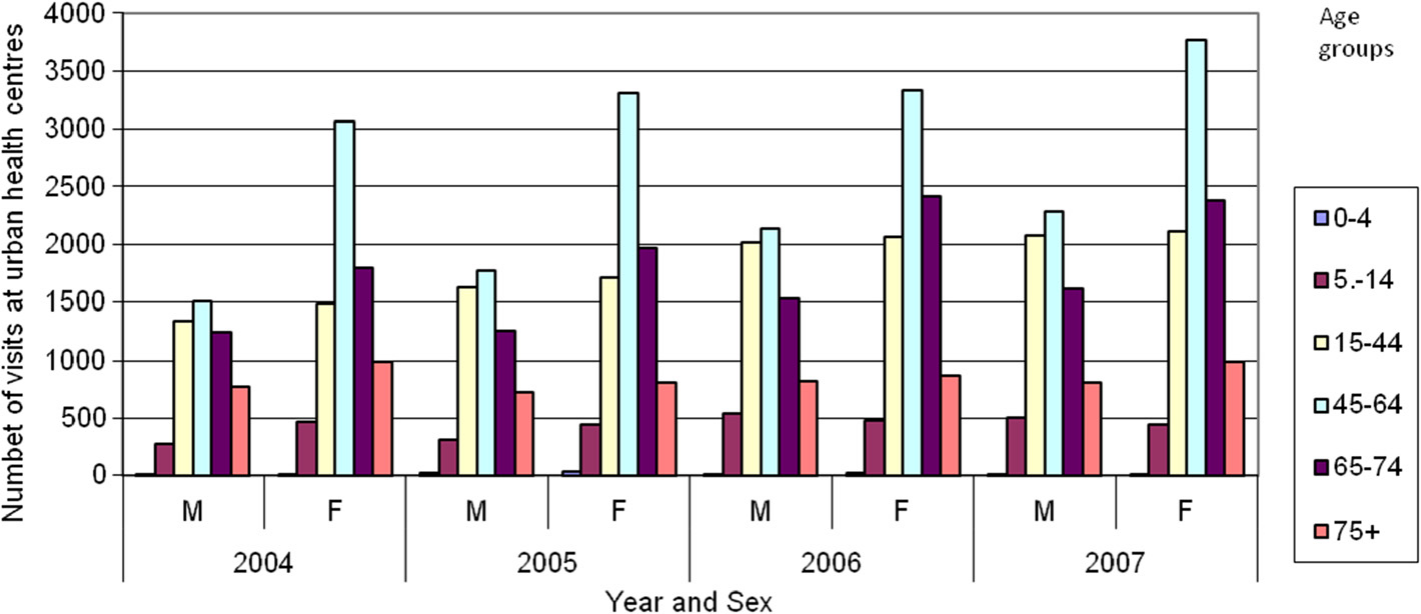
Visits in urban health centre departments by age, sex and year.

**Figure 4:**
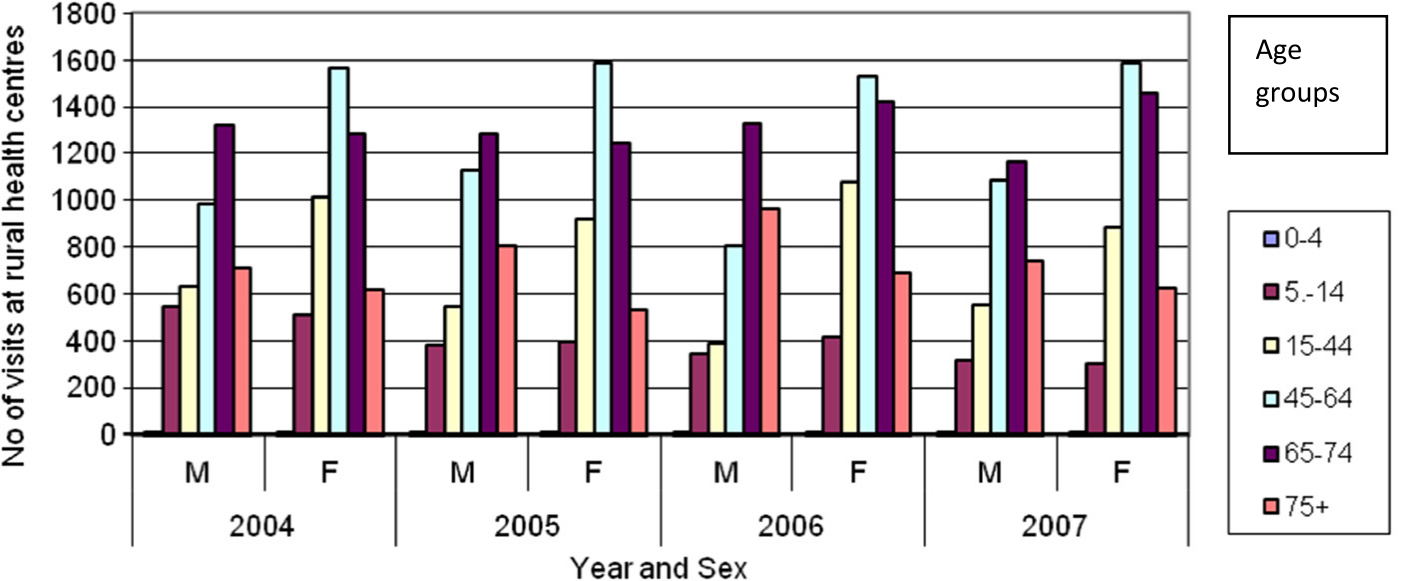
Visits in rural health centre dental department by age, sex and year.

**Figure 5:**
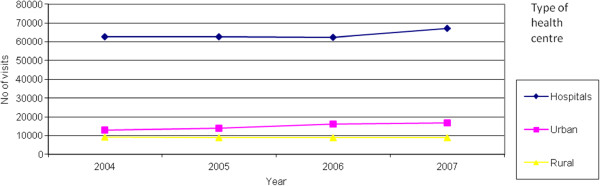
Trends in the total number of visits.

Figures 2, 3 and 4 present the number of patients who visited alternative dental health provision service places by sex, age and year. It is noticeable that women make more use of the public dental services. In both hospitals and urban health centres the age group that makes most use of the public dental services is 45–65 years, whilst in rural health centres that age group is 65–74 years. It is obvious that the patients that are visiting rural health centres are older compare to those that are visiting urban health centres or hospitals. Furthermore, as indicated in Figure 5 there was an increase in the number of visits between 2004 and 2007 both at hospitals (7.00%) and urban health centres (31.32%) but a decrease at rural health centres (5.11%). The increase in the number of visits at urban health centres was to be expected as a result of the migration of population from rural into urban areas. It was also thought that because urban health centres are located in new and modern buildings this may make it more attractive for patients to visit them although it must be clarified that all health centres are well equipped and have the same dental units and use the same materials This should be taken into consideration in the future regarding human resource planning. As far as hospitals are concerned it is worth mentioning that from 2004 until 2006 the number of visits was stable, followed by an increase in 2007. This needs to be examined further with regard to the underlying reasons and to whether it is likely to continue in the future.

**Figure 6:**
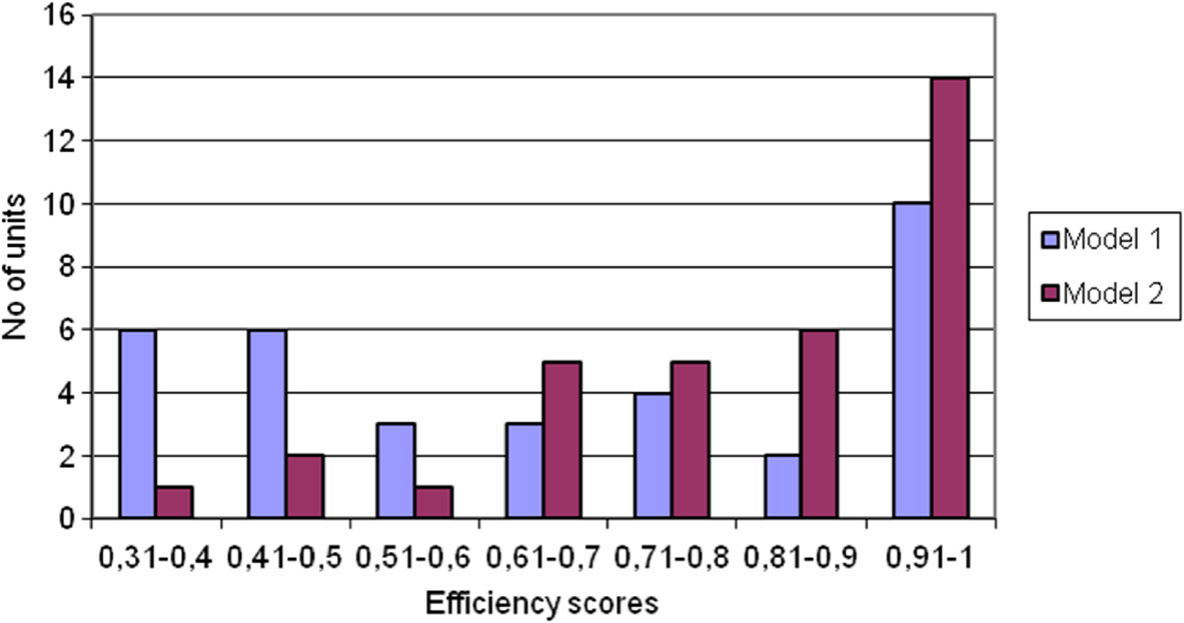
Distribution of PDS units according to their efficiency.

In the first model mean efficiency across all production units was 67.8%, ranging between 30.9% and 100%. In the second model mean efficiency across all production units was 81.2%, ranging between 39.3% and 100%. These results mean that producers could deliver the same amount of services using about 32.2% (model 1) or 18.8% (model 2) less inputs, or alternatively that with the resources used 18.8% to 32.2% more services could be produced to eliminate inefficiency. The differences observed between the models were rather expected. The lower scores in the first model are associated with the higher number of input variables, where more complexity lays the ground for greater inefficiency. The first model may better reflect the reality, as the mean cost of materials at hospitals and rural health centres corresponds to only 7% of the mean cost of the wages, whilst this rises to 9% for urban health centres. This is also indicated in Figure 6, which depicts the distribution of centres according to their efficiency score. However, looking at the lists of centres with the greatest and the lowest efficiency scores (Table 4) we notice that these are almost identical in both the models, an element which enhances the reliability of our findings.

**Table 4:** Centres with the lowest and the highest scores of TE

	***Model 1***		***Model 2***	
**A/A**	**Centre**	**Efficiency**	**Centre**	**Efficiency**
**Bottom**				
**1**	Health Centre 1 (3)	30.85	Health Centre6 (1), (3)	39.26
**2**	Health Centre 2 (3)	32.40	Health Centre4 (3)	40.90
**3**	Health Centre 3 (3)	32.41	Health Centre9 (1) (3)	45.10
**4**	Health Centre 4 (3)	32.50	Health Centre10	54.92
**5**	Health Centre 5	38.03	Health Centre5	60.51
**6**	Health Centre 6 (1) (3)	39.26	Health Centre2 (3)	62.67
**7**	Health Centre7	42.40	Health Centre11	65.90
**8**	Health Centre8 (3)	43.62	Health Centre1 (3)	68.94
**9**	Health Centre9 (1) (3)	45.10	Health Centre7	69.20
**10**	Health Centre10	45.23	Health Centre12	71.89
***Top***				
**1**	Hospital 1	100	Hospital 1	100
**2**	Hospital 2	100	Hospital 2	100
**3**	Health Centre13 (2)	100	Health Centre 13 (2)	100
**4**	Health Centre 14 (rhc)	100	Health Centre14 (rhc)	100
**5**	Health Centre15 (rhc) (2)	100	Health Centre15 (rhc) (2)	100
**6**	Health Centre16 (rhc)	100	Health Centre16 (rhc)	100
**7**	Health Centre17	100	Health Centre17	100
**8**	Health Centre18 (rhc) (1)	100	Health Centre 18 (rhc) (1)	100
**9**	Hospital 3	93	Health Centre20	100
**10**	Health Centre19 (rhc) (1)	91	Health Centre21	100

*Rhc*:rural health centres.

(1): Centres where the dentist works without an assistant.

(2): Centres where the working hours have been reduced.

(3): Centres that are staffed with senior dental officers.

In Table 4, it is notable that the list with the lowest scores includes only rural health centres, whereas the list with the high technical efficiency (TE) scores includes hospitals, urban and rural health centres. By examining carefully the rural health centres with high TE scores one can see that these centres have managed to improve their TE, either by reducing their inputs (e.g. rural health centres where the dentists work without an assistant or the working hours have been reduced) or by increasing their visits (outputs). On the other hand a lot of rural health centres that present the lowest efficiency scores are staffed with senior dental officers which are paid with higher wages, a factor that increase the inputs and in conjunction with the low outputs, decreases efficiency (Table 4).

No statistically significant difference was found in the comparison of efficiency between hospitals and rural health centres. But when we excluded the 5 rural health centres where the dentist works without an assistant then the difference in efficiency became statistically significant (Mann–Whitney test p= 0.04). Statistically significant differences were also found in the comparison of technical efficiency between urban and rural health centres (p=0.04) and between hospitals and urban health centres (p=0.01). In the regression analysis, technical efficiency was positively related (in both the scenarios/cases) with an urban location of health centers (except in the case where in the study were included the 5 rural health centers where the dentist work without assistant) and a low time per patient. According to the analysis, TE was not related to the age of dentists, the age of patients, or the age of assistants (Table 5).

**Table 5:** Tobit analysis using technical efficiency as the dependent variable

	***Tobit Model***			
	**Model 1**	**Model 1**	**Model 2**	**Model 2**
	**No of units**	**No of units**	**No of units**	**No of units**
	**(29)**	**(34)**	**(29)**	**(34)**
Constant	.665	.798	.907	.961
Location	-.136 (**)	-.128	-.161(**)	-.162
Time per patient	-.017 (*)	-.007(**)	-.010(**)	-.007(*)
Age of dentist	.006	.010	.004	.005
Age of patient	.017	-.016	-.004	-.0002
Age of assistants	.002		.002	
*ΤΟΒΙΤ (pseudo R*^*2*^*)*	1.49	0.52	1.24	0.95

(*) p<.0.01.

(**) p<0.05.

## Discussion

The results show that women make more use of the PDS in Cyprus. This finding is in agreement with the findings of other studies showing that women tend to use health services more frequently [9-12]. It is also of note that only a small proportion of children make use of the PDS^b^. This should be taken into consideration, as our new National Health Care System is planning to provide only preventive care to children up the age of 16. Despite the increase in the proportion of patients using the PDS from 2004 to 2007, still only 10% of the population does so. The reasons for this should be further investigated, because it is an important factor that affects efficiency (e.g. what are the reasons that motivate people not to use the PDS, despite being eligible, but to prefer the private sector: convenience, better working hours in the private sector since PDS do not operate in the afternoon, not having to wait, perceived better quality of care, variety of the offering care as PDS do not offer fixed prosthetics and orthodontic service etc.). Also the needs assessment of the oral health status of people living in Cyprus should be updated as the last one was carried out 20 years ago.

Differences were also observed in the age profile of patients visiting the various health centres as well as the type of the offering care. It is therefore necessary to further research and evaluate if the offering care meets the needs and the expectations of the people and whether adds to the improvement of the level of oral health of people living in Cyprus.

Regarding efficiency, the findings indicate that the choice of input selection affects the efficiency scores. However, it seems that many PDS are not performing as well as they could, as the average score lies between 68% and 81%. This finding agrees broadly with the results of previous studies in Finland [10-13] and in the UK [14], although a Norwegian study showed much better efficiency in the public dental care of children in Norway, because of cost containment and adjustments of staff to improvements in children’s dental health [15].

The second stage of our analysis revealed two factors contributing to TE: urban area and lower time spent per patient. These results are in agreement with other studies. It seems that people living in or close to urban areas have easier access to the PDS. They are also more health conscious, have a different epidemiological profile and, in conjunction with various influencing socio-demographic factors such as age and education, they make greater use of health services. The results of our study showed that the patients that are visiting rural health centers are older and may visit a dentist only for emergency situation when the only treatment that can be provided is the extraction. This is supported by the data provided by the PDS which they show that extractions represent 23% of the offering secondary care in rural health centers compare with 15% in urban health centers and 19,5% in hospitals. Besides 9% of the patients are visiting rural health centers because of the presence of abscess (an emergency situation) compare with 7% in hospitals and 6% in urban health centers [16].

Regarding time spend per patient Sintonen showed [17] that the number of hours spent at work without treating patients was the most important factor explaining the 14% lower productivity of public compared to private dentists in Finland. In her study Widstrom showed [18] that technical inefficiency was markedly and positively associated with a high number of dental visits.

Our study did not find any significant relation between TE and the age of dentists. This finding is in agreement with the results of a study conducted in Finland [17] and another conducted in Norway (Wang 1994) [19] which indicated that the professional experience of the dentist did not affect productivity. However, other studies found opposite results. As Gray mentions [20], dentists have an unusual age learning profile in relation to those of comparable professional groups. Dentists tend to peak midway through working life and decline towards retirement. Scheffler and Kushman (1977) [21] identified peak output at around age 45 in the US, with a fall of 11% by age 60. Increasing age per se, as Sheiman noted [22], reduces productivity through reduced manual dexterity. In addition, age is thought to be inversely related to willingness to innovate, both technologically and organizationally. Brennan in a review of dental productivity found that young dentists work less efficiently than older ones [23].

The most interesting policy question is how the observed inefficiency in some units could be improved. Better use of human resource management (e.g. in remote health centers or centers with less visits/workload the dentists should work without an assistant in order to reduce inputs and improve efficiency) and also measures to improve staff performance should be employed. In addition, a survey should be undertaken to identify barriers that limit PDS use, so as to address capacity and demand issues. However, whilst efficiency is a predominant criterion for resource allocation it is not the only one. The other equally important criterion is equity. Therefore, the solution of shutting down the rural health centers that serve only a small number of patients is undesirable for populations with already limited health care options.

Although the material used was representative of public dental care in Cyprus and the quality of data was satisfactory, there remained a number of limitations in the study. First of all, we did not investigate the quality of the treatment offered, assuming that this was similar in all centers. However, the fact that in some health centers (regardless of the small number of patients in these centers) the dentists are working without assistant, this can affect negatively the quality of the offering care. Second, efficiency should also include measures of final outputs rather than the intermediate outputs that were used in this study. It is possible, for example, that slower but more considered care could be more efficient in terms of health outcomes that it seems in terms of intermediate outcome. Also there are other variables that may affect efficiency - such as specialization of the personnel - that have not been addressed in this study. These are areas to be addressed in future research efforts.

## Conclusions

This is the first study of its type in Cyprus. It found that public dental services are used more by women, and by middle and older aged patients. Although there is in increasing trend in the usage, still only a small percent of the population make use of them. In terms of their efficiency there is significant room for improvements that will result in better use of the resources and maximisation of the services delivered to the public. Urban areas and lower time per patient are predictors of increased efficiency. Based on this study, measures to improve the efficiency of the PDS should be developed and implemented. In addition, there is a need for further research in terms of interviews, surveys of the staff and the patients regarding the type and the quality of the offering care and whether this satisfies the patients’ needs and expectations and also whether this adds to the improvement of the level of oral health of people living in Cyprus. With these changes in place, this study should then be repeated in the future to provide evidence for improvements in the practice and efficiency of the PDS.

## Endnotes

^a^People live in rural areas are older and belong to lower socioeconomic classes compare to people live in urban areas as most of them are workers and farmers. Besides according to the national survey conducted in 1993, adults living in rural areas had statistically greater level DMFT and more treatment needs than those living in urban areas [24].

^b^It has to be mentioned that the PDS are offering oral health education at schools to all the children age 6–12 years old. Besides with the help of 4 mobile units are visiting elementary schools which are situated in remote areas with limited access to dental care and significant oral health problems and are offering free treatment and prevention.

## Competing interests

The authors declare that they have no competing interests.

## Authors’ contributions

NM, NP, MT designed and coordinated the study. CC, VF undertook the analysis of data and the writing of the manuscript. All authors read and approved the final manuscript.

## Pre-publication history

The pre-publication history for this paper can be accessed here:

http://www.biomedcentral.com/1472-6963/13/420/prepub
